# Tuning apparent friction coefficient by controlled patterning bulk metallic glasses surfaces

**DOI:** 10.1038/srep39388

**Published:** 2016-12-19

**Authors:** Ning Li, Erjiang Xu, Ze Liu, Xinyun Wang, Lin Liu

**Affiliations:** 1 School of Materials Science and Engineering, Huazhong University of Science and Technology, Wuhan 430074, PRC; 2 State Key Laboratory of Solidification Processing, Northwestern Polytechnical University, Xi’an 710072, PRC; 3 Department of Engineering Mechanics, School of Civil Engineering, Wuhan University, Wuhan 430072, PRC

## Abstract

Micro-honeycomb structures with various pitches between adjacent cells were hot-embossed on Zr_35_Ti_30_Cu_8.25_Be_26.75_ bulk metallic glass surface. The effect of pitch geometry on the frictional behavior of metallic glass surface was systematically investigated. The results revealed that all textured metallic glass surfaces show a reduction in friction coefficient compared to smooth surface. More intriguingly, the friction coefficient first decreased and then increased gradually with increasing pitches. Such unique behavior can be understood fundamentally from the perspective of competing effects between contact area and local stress level with increasing pitches. This finding not only enhance the in-depth understanding of the mechanism of the significant role of surface topography on the frictional behavior of metallic glass surface, but also opens a new route towards other functional applications for bulk metallic glasses.

Bulk metallic glasses (BMGs) are a fascinating class of metallic alloys with an isotropic amorphous structure that endows them with a plethora of outstanding properties such as close to theoretical strength, high elasticity, high hardness, appreciable toughness and superior corrosion resistance^1^,^2^,^3^. Whereas the high strength and poor plasticity have limited the processing of metallic glasses at ambient temperature. The nature of amorphous structure with the existence of a supercooled liquid regime (SCLR) between the glass-transition temperature (*T*_*g*_), and the crystallization temperature (*T*_*x*_) allows thermoplastic forming (TPF) of BMGs. TPF provides a promising technique for precise net-shaped versatile geometries comprising of micro- and nano-sized features, exhibiting an alluring prospect in micro- and nano-engineering applications^4^,^5^,^6^,^7^,^8^.

The hot-embossed metallic glass nanowire architectures with high aspect ratios (>200)^7^,^9^, present superb durability combined with high electrocatalytic activity toward CO, methanol, and ethanol oxidation, displaying vast potential in areas such as energy conversion/storage and sensors^10^. This motivates the generation of first functional proton exchange membrane micro fuel cells (MFCs) with high-surface area catalytic and gas flow field components made from BMGs. Such MFCs have been identified as promising alternative power sources for portable electronics^11^. The hot-imprinted nano-patterns on metallic glasses surfaces also present potential applications in anti-reflection materials, cell culture medium for bio-chips, electrode materials, hologram technology, and next generation ultra-high density of information data storage material^12^. The embossed hierarchical structures integrating nano-, micro- and macro-sized features, show versatile functions in wetting, cellular response, electrochemical activity and optical devices^13^. The thermoplastic formed micro-patterns, for example, micro-lens arrays can be utilized as aspheric lens^14^; micro-channel geometries can be employed as fuel cell interconnect plates^15^, and robust tools to replicate the micro patterns by micro-imprinting of the Polymethyl methacrylate (PMMA)^16^,^17^,^18^. The 3D micro-structures that were hot-embossed from BMG, exhibit some possible applications such as high-Q (lightly damped) micro-resonators for the telecom industry, microsurgical tools and devices, microscale motors and transmission components^19^. Our recent researches have shown that the hot-patterned metallic glass surface display great potential in the fabrication of superhydrophobic surfaces (contact angle greater than 150°) with long lifespan in service^20^,^21^.

It is worth noting that these patterned micro-scale textures on solid surfaces should change the interfacial tribology properties, and consequently affect the frictional behavior and wear process. For example, micro-patterned tool’s surface often reduces the coefficients of friction, cutting force and tool wear^22^, nano-patterned channel boundaries facilitate the low-friction flows of liquid^23^. In general, the crucial role of surface textures as oil pockets to reduce friction and the assistance in tripping off wear debris^24^,^25^,^26^. Recently, Rashwan^27^ reported the effect of surface texturing on tribological performance, and regarded that real area of contact and material properties are two key factors that control friction. Whereas, frictional anisotropy cannot be ignored in understanding the underling mechanisms in crystalline materials^28^. For BMGs with isotropic microstructure, previous literatures^29^,^30^ reported that the tribological performances are correlated to abrasive conditions and testing parameters, such as loading, sliding speed, sliding distance, lubricating condition, friction mode, testing environment, etc. The question then arises as what kinds of pattern on BMGs’ surface are better for friction reduction, and what is the underlying origin for the intrinsic effect of surface patterns on tribological performance? To investigate these questions, we designed a systematic experiment, and found that the coefficient of friction (COF) decreases first, and then increases gradually with increasing pitches between adjacent cells (*P*). Such a phenomenon can be understood fundamentally from the perspective of varying contact area and local stress level with increasing pitches.

## Results

**Figure 1:**
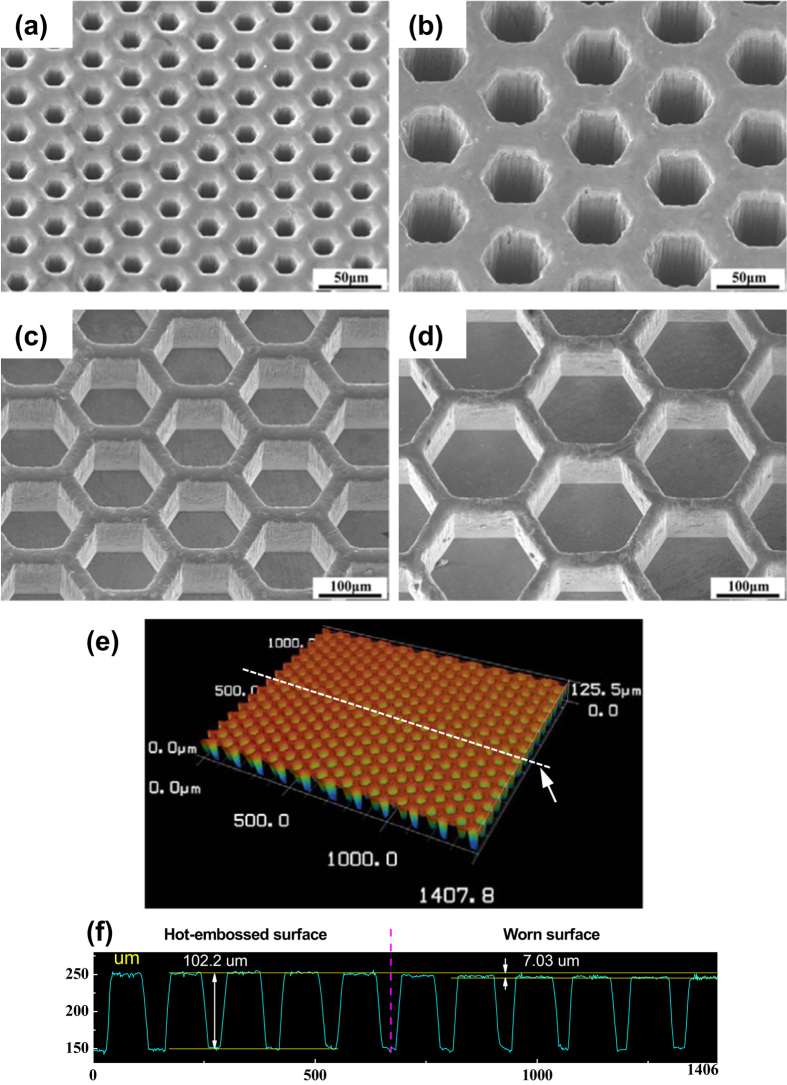
Hot-patterned micro-honeycomb structures with various pitches, (**a**) 35.5 μm; (**b**) 75.5 μm; (**c**) 155 μm; (**d**) 195 μm. (**e**) the 3D profile of honeycomb pattern with pitch of 75.5 μm, (**f**) the line scanning profile of adjacent cells, indicating the average height of 102.2 μm of the pattern.

Figure 1a to d show the SEM micrographs of the hot-embossed honeycomb patterns with various pitches between adjacent honeycomb cells (*P* = 35.5 μm, 75.5 μm, 155 μm and 195 μm, respectively). To reveal the filling depths after thermoplastic embossing, the 3D and cross section profile of the honeycomb patterns for *P* = 75.5 μm were examined through laser scanning confocal microscope (Keyence VK-X200 series) and shown in Fig. 1e, which reveals an average height of about 102.2 μm of the pattern (Fig. 1f).

**Figure 2:**
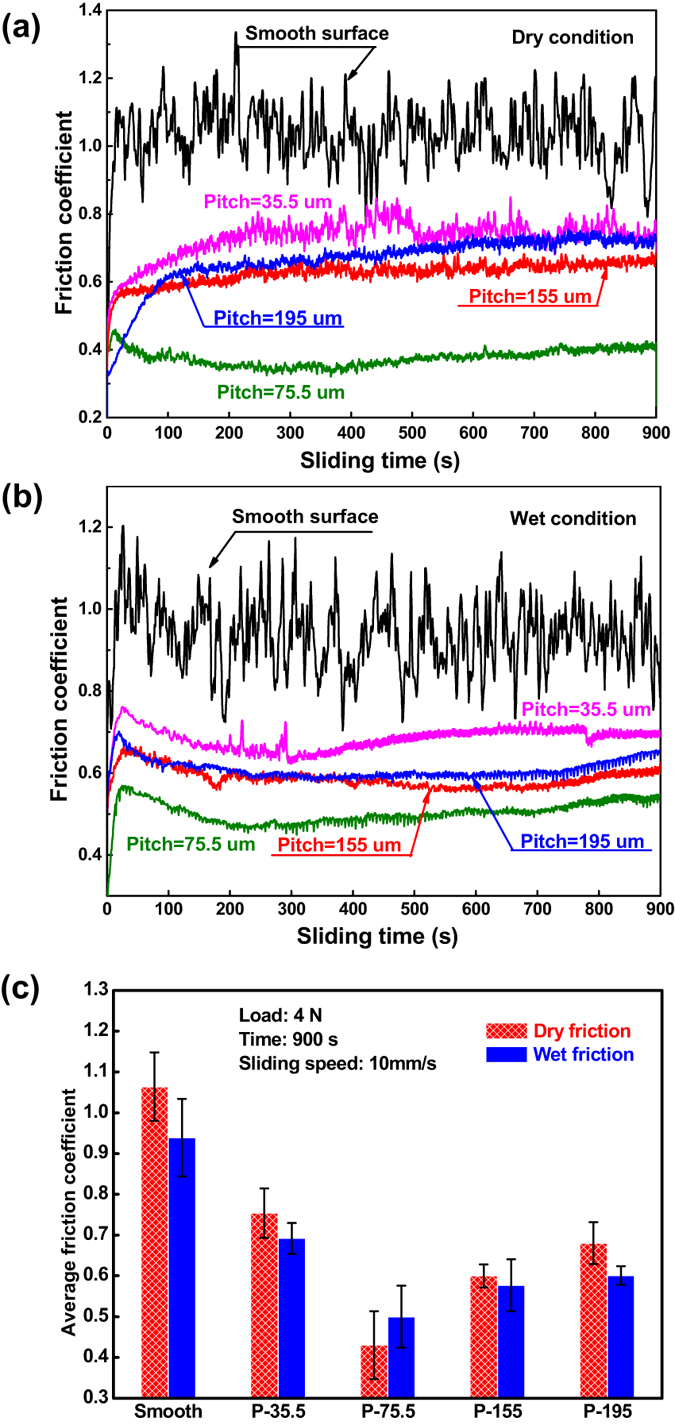
(**a**,**b**) Friction coefficient-sliding time curves of the hot-patterned Zr-based BMG surfaces under dry and wet conditions, respectively; (**b**) the corresponding average friction coefficients.

Figure 2a displays the friction coefficient (*μ) vs.* sliding time curves of the Zr-based BMG surfaces under dry condition. It is clear that the value of friction coefficient is about 1.06 ± 0.08 for the smooth surface, while for the hot-patterned surface, the coefficient of friction decreased systematically (*μ* < 0.8). For example, *μ* decreases to 0.75 ± 0.06 at pitch of 35.5 μm. When pitch increases to 75.5 μm, *μ* reduces further to minimum value of 0.43 ± 0.08. However, *μ* increases up to 0.60 ± 0.03 and 0.68 ± 0.05 when pitch increases to 155 μm and 195 μm, respectively. The similar phenomenon can also be observed under wet condition, as shown in Fig. 2b. To clearly distinguish the effect of pitches and experimental conditions on the tribology property, the average friction coefficients of all surfaces with various pitches are summarized in Fig. 2c. For most pitches, the value of *μ* under dry friction is larger than that suffered wet condition. Whereas in both cases, a U- or V-like shape can be observed, namely the friction coefficient decreases first, and then increases gradually with increasing pitches.

**Figure 3:**
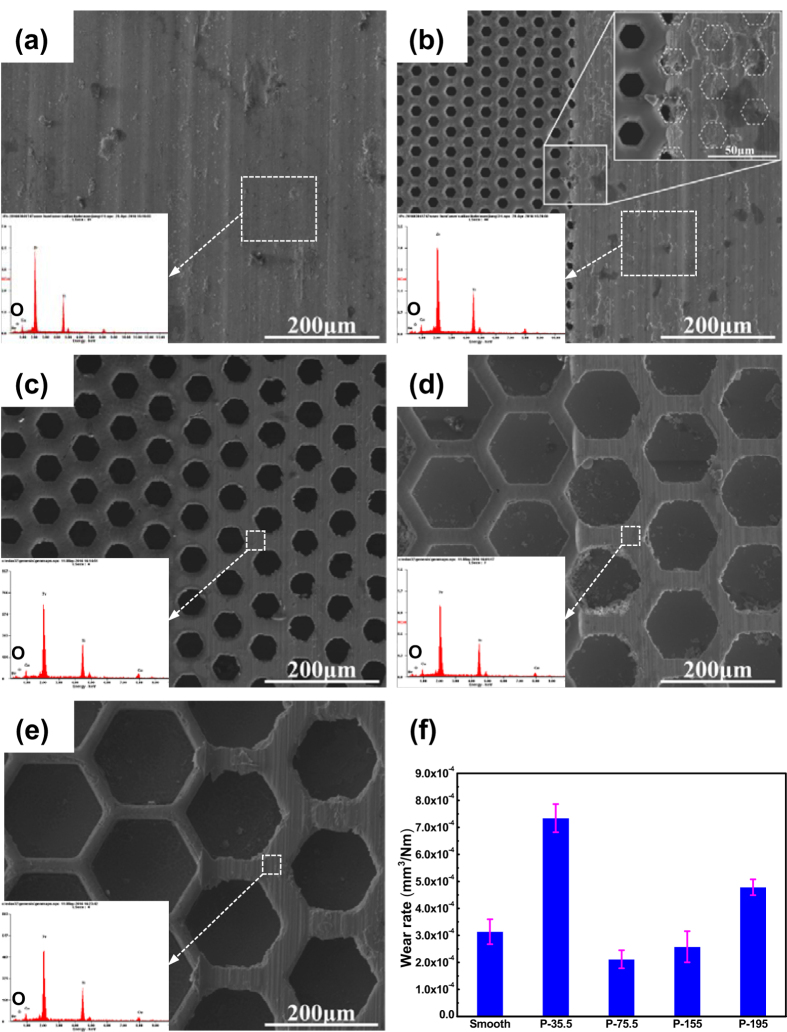
SEM micrographs of the worn surfaces, the inset shows the elements distribution in worn surface (dashed square frame): (**a**) smooth surface; (**b**–**e**) surface with p = 35.5 μm, 75.5 μm, 155 μm and 195 μm, respectively; (**f**) the corresponding wear rates.

To further analyze the difference of tribology properties among Zr-based BMG surfaces with various pitches, the surface topographies of BMG specimens after dry friction were selected and characterized by SEM, as depicted in Fig. 3a to e. From which parallel and abrasive grooves along sliding direction are observed in all worn surfaces, indicates the generation of plastic deformation during friction. It can be observed that on hot-patterned BMG surface with pitch of 35.5 μm, all hexagonal cellular of the worn surface were covered by the debris (see the insert in Fig. 3b), indicates a serious plastic deformation. While for patterned BMG surface with pitches ranging from 75.5 μm to 195 μm, the surface topographies are similar, as observed in Fig. 3c to e. The corresponding wear rate (*w*_*r*_) was calculated based on.


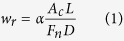


In which *α* is the areal fraction of the surface patterns, *A*_*c*_ is the cross-sectional area of worn track (product height and width obtained in scanning profile as shown in Fig. 1f), *L* is the length of worn track, *F*_*n*_ = 4N is the normal force and *D* = 9 m is the sliding distance. The application of equation 1 to the data (listed in Table 1) yields calculated wear rates, as illustrated in in Fig. 3f. From which a U- or V-like shape of wear rate can also be observed, wherein the patterned surface with pitch of 75.5 μm exhibits the minimum value of *w*_*r*_, similar to the average friction coefficient as described in Fig. 2c.

It is generally accepted that most of frictional work during the wear process is converted into heat, which in turn, raises the interface temperature, modifies properties of sliding surfaces such as forming oxide layer or even melting interfacial materials^31^,^32^,^33^,^34^. Here, The maximum contact temperature rise Δ*T*_max_ can be estimated by the following equation^35^,


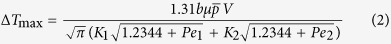


where *b* is the radius of contact circle (mm), *μ* is the friction coefficient, 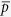 is the mean contact pressure (MPa), *V* is the sliding velocity (m/s), *K*_1_ and *K*_2_ are thermal conductivities (W/(m K)) of Si_3_N_4_ and Zr-based BMG, respectively, and *P*_*e*1_ and *P*_*e*2_ are Peclet numbers. Here,


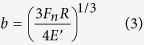



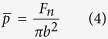


in which


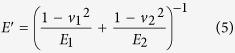



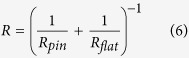



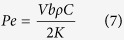


where *ρ* is the density (kg/m^3^), *C* is the specific heat (J/Kg·K), *R*_*pin*_ = 1.3 mm is the radius of the Si_3_N_4_ pin, *R*_*flat*_ → ∞, *E*_1_, *E*_2_ is elastic modulus (GPa) and *v*_1_, *v*_2_ is poisson’s ratio of Si_3_N_4_ and Zr-based BMG, respectively. According to equation 2 and parameters listed in Table 2, Δ*T*_max_ = 10.8 °C is calculated, which is far lower than the glass transition temperature (*T*_*g*_ = 307 °C) and the crystallization temperature (*T*_*x*_ = 448 °C), therefore cannot induces the crystallization during wear process. This result was further demonstrated through energy dispersive X-ray (EDX), the scarce oxygen peak is observed in the insert in Fig. 3a to e, and the low oxygen content is depicted in Table 3.

## Discussion

The above experimental results clearly reveal reduced coefficients of friction for the patterned Zr-based BMG surfaces under dry and wet conditions, as compared with the smooth surface. In general, surface patterns or asperities were regarded as the oil pockets to reduce friction in two ways, one is the secondary lubrication effect, namely the surface textures act as a secondary oil source thus supplying lubricant continuously to the contact area to reduce friction and retard galling. The other is hydrodynamic effect wherein the surface asperity builds an additional hydrodynamic pressure which separates both rubbing surfaces, increases the oil film thickness and enhances load bearing capacity^24^,^25^. This is the main reason for what we observed in Fig. 2c, wherein the friction coefficient under wet condition is lower than that under dry condition. However, the friction coefficient is sensitive to the variation of pitch in both conditions, demonstrates the possibility to tuning apparent tribological performance by surface patterning.

The patterned surfaces were usually considered as providing traps for wear debris in dry contacts, which improves surface’s wear resistance and fatigue life. Though Rashwan^27^ regarded that real area of contact and material properties are two key factors that control friction, but for BMGs with isotropic microstructure, the physical origin for the intrinsic effect of surface topographies on friction remains is still unclear. In microscale, as proposed by Bowden *et al*.^36^, friction force (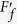) is directly proportional to the real contact area (*A*, the quantitative measurement is rather difficult) between two sliding surfaces, *i.e.* 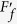 = *τA*. Where *τ* is the shear strength of the contact. In general, *A* is proportional to the apparent contact area (*S*_*a*_) but much less than *S*_*a*_ because real surfaces always possess some degree of roughness and contact only occurs at or near the peaks of these contacting asperities. In our case, *S*_*a*_ = *αS*, where *S* is the apparent area of the slier (Si_3_N_4_ pin), so the friction is


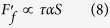


From Table 1, the areal fraction of the surface patterns (*α*) decreases with the increase of pitch, which evidently results in the reduction of friction coefficient (is proportional to 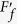) according to equation 8. However, the friction coefficient not always decreases with increasing pitch, but exhibits a minimum value at pitch of 75.5 μm, and then increases gradually when pitch increase from 75.5 μm to 175 μm, displays an inconsistent result, as what was observed in Fig. 3c.

The increasing pitch induced the reduction of *α*, on the other hand, raises the contact stress (*F*_*n*_/*A*) significantly. For example, the calculated contact stress is about 0.75 MPa on smooth surface, but it increases to 9.4 MPa on patterned surface with pitch of 195 um. Considering that the real contact in the microscale originated from asperities. The larger contact stress will result in higher penetration of hard asperities in the harder surface (Si_3_N_4_ pin here) into the relatively soft substrate, which can significantly enhance the ploughing effect in macroscale and contribute to the measured friction. By assuming a spherical asperity and based on Hertz model, the penetration depth (*h*) of the asperity scales with normal force on the asperity (*F*_*s*_) as 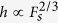
37, and the ploughing area is proportional to *h*. In our case, 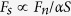, hence we obtain the friction resulting from ploughing effect in macroscale as,


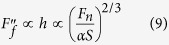


According to Amontons’ law^37^, the friction force (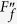) increases linearly with the normal force (*F*_*n*_) in macroscale. Considering the equivalence of describing friction behavior from macroscale and microscale on the basis of equations 8 and 9, the friction coefficient (*μ*) can be finally obtained as,


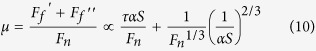


**Figure 4:**
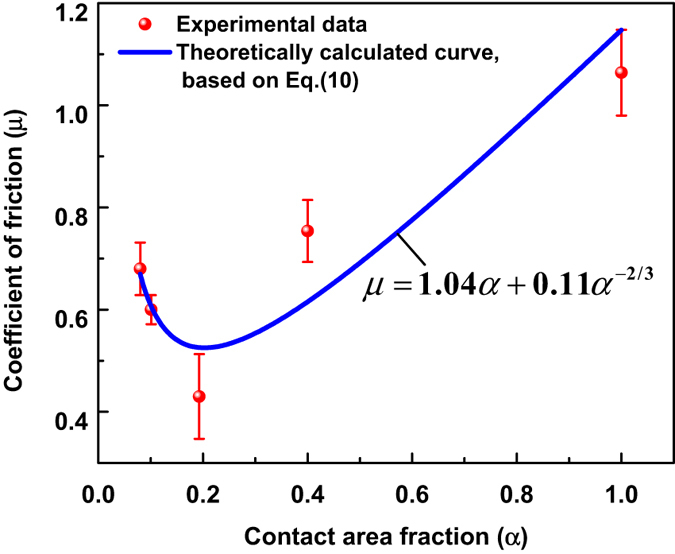
The experimental and theoretically calculated coefficients of friction with the contact area fraction, blue line represents that the calculated data fits well with the experimental data.

According to equation 10, the theoretical curve of coefficient of friction (*μ*) with the contact area fraction (*α*) is fitted as depicted in Fig. 4, which matches well with the experimental results, demonstrating that the observed concave shape of *μ* with the decrease of *α* (corresponding to increasing pitch as listed in Table 1) originates from the competition between contact friction and ploughing effect on the microscale.

According to the adhesion theory of friction, the average normal stress in the real contact area (only occurs at or near the peaks of asperities) can easily exceed the material hardness, and each asperity contact is viewed as a plastic indentation^38^. Therefore, the increasing pitch induced the raising of contact stress, which aggravates the plastic deformation, resists the relative motion of two surfaces and results in an increase of friction coefficient. Through the above analysis, we can understand fundamentally that the competition between increasing pitch-induced the reduction of contact area and the increase of contact stress, responsible for the U- or V-like shape change of friction coefficient variation with increasing pitch, as illustrated in Fig. 2c.

## Conclusions

In summary, Zr-based bulk metallic glass surfaces with honeycomb microstructure were successfully fabricated through thermoplastic forming and the intrinsic effect of surface pattern on friction characteristics was investigated systematically. By comparison with the smooth surface, the friction coefficient of the patterned surfaces is lower, and a minimum value was observed at pitch of 75.5 μm. The U- or V-like shape change of friction coefficient with increasing pitch can be understood well by considering the competition between increasing pitch-induced the reduction of contact area and the increase of contact stress.

## Experimental methods

### BMG sample preparing

A Zr_35_Ti_30_Be_26.75_Cu_8.25_ (at. %) BMG system was considered for this study owing to its excellent anti-oxidation capability, large supercooled liquid region (SCLR, over 141 K) and good microformability^3^,^8^,^39^,^40^,^41^. The BMG plates with dimension of 67 mm × 14 mm × 2 mm were fabricated by arc-melting a mixture of pure Zr, Ti, Be and Cu metals (purity > 99.5%) under a Ti-gettered argon atmosphere, followed by jet casting into a cooper mould. The glassy structure of the as-cast alloy was verified by X-ray diffraction (XRD, Philips χ’ Pert Pro) and transmission electron microscopy (TEM, Jeol-2010). The thermal response was determined by differential scanning calorimetry (DSC, TA Q2000) at a heating rate of 20 K min^−1^, showing a glass transition temperature (*T*_*g*_) of 307 °C with a wide supercooled liquid region of 141 °C. The isothermal crystallization experiments were carried out by DSC at various temperatures in supercooled liquid region, which reveals the incubation time is around 1 min at 450 °C, 150 min at 390 °C and more than 300 min at 370 °C for crystallization. The results are identical to the time–temperature–transformation (TTT) diagrams measured by Duan *et al*.^39^. The BMG samples used for the hot-imprinting experiment with dimension of 10 mm × 10 mm × 2 mm were prepared by wire-cutting from the as-cast amorphous sheets and the two parallel surfaces were polished to mirror-like finish to eliminate surface oxidation and defects. The silicon master moulds with various honeycomb structures (the wall thickness of 8 μm, a depth of 100 μm and pitches, *P*, ranging from 35.5 to 195 μm) were designed and produced through deep reactive ion etching^20^.

### Thermoplastic microforming of BMG

The hot-embossing experiments for the Zr-based BMG were performed at the temperature of 390 °C (~1.3 *T*_*g*_) to avoid any crystallization and strain rate of 1 × 10^−3^ s^−1^ using a REGER machine (RGM-4050) equipped with an air furnace. In order to shorten the time for heating the sample, the load train was preheated to the test temperature and held for 30 min to stabilize the temperature, the BMG specimen was stacked on silicon master mould and then rapidly placed into the load train with a preload of about 100 N and held for another 3 min to attain thermal equilibrium. The fluctuation of temperature in the furnace during testing was about ±1 K. After the hot-embossing process, the embossed metallic glass part was separated from the silicon mould by etching away the silicon in a KOH bath for 2 h at 80 °C. Micrographs of the hot-embossed Zr-based BMG with microchannel structures were observed by scanning electron microscopy (SEM, Qutanta 400), and the filling depths (*h*) were measured according to the side morphologies.

### Friction experiments of patterned BMG surface

**Figure 5:**
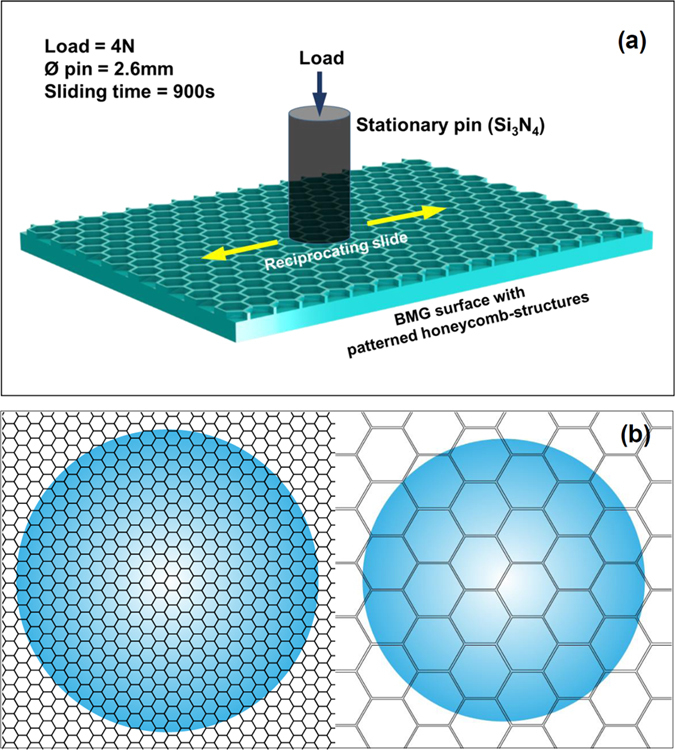
(**a**) Schematic illustration of the friction experiment on the hot-patterned Zr-based BMG surface. (**b**) top view of the friction on the patterned surfaces with various pitches.

The frictional experiments were carried out on the hot-patterned BMG surfaces by pin-on-flat reciprocating tests on a Bruker UMT-3 Tribometer with a Si_3_N_4_ pin (Ø2.6 mm), as illustrated in Fig. 5a. Here a 4N force was applied, slide reciprocally with speed of 10 mm/s and time of 900 s in-air temperature at dry condition and water was acted as lubricant at wet condition. Figure 5b illustrates the top view of the friction on the patterned surfaces with various pitches. The morphologies of the hot-embossed and worn BMG surfaces were subsequently characterized by SEM/EDX and Laser Scanning Confocal Microscope (LSCM, Keyence VK-X200 series).

## Additional Information

**How to cite this article:** Li, N. *et al*. Tuning apparent friction coefficient by controlled patterning bulk metallic glasses surfaces. *Sci. Rep.* **6**, 39388; doi: 10.1038/srep39388 (2016).

**Publisher's note:** Springer Nature remains neutral with regard to jurisdictional claims in published maps and institutional affiliations.

## Figures and Tables

**Table 1:** Parameters of the Zr-based BMG surfaces under dry friction.

Pitch (μm)	Height (μm)	Measured (°)^20^	Areal fraction of the surface patterns, *α*	*A*_*c*_ (mm^2^)	*L* (mm)
0	0	98.8 ± 2.6	1	2.05 × 10^−3^	5.5
35.5	100	126.0 ± 1.8	0.4	1.32 × 10^−2^	5
75.5	100	138.5 ± 1.3	0.193	7.90 × 10^−3^	5
155	100	153.5 ± 1.8	0.101	1.84 × 10^−2^	5
195	100	149.3 ± 0.9	0.08	4.31 × 10^−2^	5

**Table 2:** Parameters of Si_3_N_4_ and Zr-based BMG.

	Si_3_N_4_	Zr-based BMG
*E*, Modulus of Elasticity (GPa)	308^35^	86.9^39^
*ρ*, Density (Kg/m^3^)	3180^35^	5396*
*υ*, Poisson’s ratio	0.26^35^	0.37^39^
*K*, Thermal Conductivity (W/m·K)	16.7^35^	~5.83^42^
*C*, Specific heat (J/Kg·K)	—	~519*

^*^Material properties (measured).

**Table 3:** Oxygen content on the origin and worn surfaces.

Pitch (μm)	O (at. %)		
	Origin	Wet	Dry
0	02.61	05.09	04.64
35.5	31.79	06.65	04.54
75.5	28.88	04.80	04.99
155	20.94	02.33	07.62
195	35.68	01.67	05.61
